# Large-Scale Variations in Lumber Value Recovery of Yellow Birch and Sugar Maple in Quebec, Canada

**DOI:** 10.1371/journal.pone.0136674

**Published:** 2015-08-27

**Authors:** Mariana Hassegawa, Filip Havreljuk, Rock Ouimet, David Auty, David Pothier, Alexis Achim

**Affiliations:** 1 Department of Wood and Forest Sciences, Laval University, Quebec City, Quebec, Canada; 2 Forest Research Division, Ministry of Forests, Wildlife and Parks, Quebec City, Quebec, Canada; DOE Pacific Northwest National Laboratory, UNITED STATES

## Abstract

Silvicultural restoration measures have been implemented in the northern hardwoods forests of southern Quebec, Canada, but their financial applicability is often hampered by the depleted state of the resource. To help identify sites most suited for the production of high quality timber, where the potential return on silvicultural investments should be the highest, this study assessed the impact of stand and site characteristics on timber quality in sugar maple (*Acer saccharum* Marsh.) and yellow birch (*Betula alleghaniensis* Britt.). For this purpose, lumber value recovery (LVR), an estimate of the summed value of boards contained in a unit volume of round wood, was used as an indicator of timber quality. Predictions of LVR were made for yellow birch and sugar maple trees contained in a network of more than 22000 temporary sample plots across the Province. Next, stand-level variables were selected and models to predict LVR were built using the boosted regression trees method. Finally, the occurrence of spatial clusters was verified by a hotspot analysis. Results showed that in both species LVR was positively correlated with the stand age and structural diversity index, and negatively correlated with the number of merchantable stems. Yellow birch had higher LVR in areas with shallower soils, whereas sugar maple had higher LVR in regions with deeper soils. The hotspot analysis indicated that clusters of high and low LVR exist across the province for both species. Although it remains uncertain to what extent the variability of LVR may result from variations in past management practices or in inherent site quality, we argue that efforts to produce high quality timber should be prioritized in sites where LVR is predicted to be the highest.

## Introduction

Forestry practices in the northern hardwood forests have for many decades favoured the selective harvesting of the most valuable trees available, which has resulted in the general depletion of the resource [1–3]. To reverse this trend and promote forest restoration, new stem marking rules were introduced in the public forests of Quebec, Canada, to ensure harvesting of low-vigour trees in selection cuts [4, 5]. However, the current state of these forests can affect the financial applicability of this silvicultural system [6]. The often low-quality wood obtained from low-vigour trees and the reduced demand for pulpwood limit the capacity to apply such forest restoration measures in northern hardwood forests.

The search for solutions to this problem has mainly focused on improving the stem selection process during harvesting operations. Pothier et al. [6] argued that among non-vigorous stems expected to die before the next scheduled cut, those that have maintained a high quality should be selected for harvest. This could be achieved by establishing a marking priority for non-vigorous trees exempt from cracks and external signs of fungal infections, and with a diameter at breast height approaching 40 cm [7]. In addition to applying such rules within a given cutblock, the strategy should also consider the variability among sites, so that restoration measures can be applied where the potential return is the highest [8]. However, evaluating the propensity of a site for the production of high quality timber is complicated by 1) the fact that the characteristics of the current resource are influenced both by the intrinsic characteristics of the site and by the effects of past disturbances, particularly high-grading practices and 2) the multiple potential definitions for ‘timber quality’.

Whereas the long-term effects of various silvicultural scenarios have been documented to some extent in the literature [9, 10], there is much less information about how site characteristics might affect the quality of timber from northern hardwoods. In one of the few studies available on the subject, Havreljuk et al. [11] described the regional pattern of variation in the proportion of discoloured heartwood in yellow birch (*Betula alleghaniensis* Britt.) and sugar maple (*Acer saccharum* Marsh.). Although tree size and growth rate descriptors accounted for most of the regional variability, results also showed a negative correlation between the red heartwood proportion in sugar maple stems and extreme minimum temperature at a given site. For yellow birch, Gagné et al. [12] found a correlation between stem quality and the mean annual precipitation. Such correlations between wood properties and site characteristics have also been observed in other species and forest types [13–16]. Despite the fact that true causal links between site characteristics and wood properties have yet to be elucidated, these studies tend to confirm that some sites have a higher potential than others for producing wood of high quality in a given species. However, among the several factors known to induce variations in wood quality within and between stems, the effects of site characteristics arguably remain the least documented [17].

The concept of timber quality implies the association with a specific end-use [18, 19]. In northern hardwoods, the production of sawn boards for the subsequent manufacture of ‘appearance’ wood products, such as flooring and furniture, is usually the primary processing option that generates the most monetary value [7]. Consequently, lumber value recovery (LVR) may be used as an indicator of timber quality. First described by McCauley and Mendel [20] in the context of sawmilling studies, LVR is essentially an estimation of the summed monetary value of boards contained in a unit volume of round wood. Because stumpage prices are also determined per unit volume of round wood, LVR can be considered as a tangible estimate of ‘value’, as perceived by a sawmill purchasing logs and selling boards on the National Hardwood Lumber Association market [21]. An advantage of this metric is that it is less dependent on tree size than the summed value of boards contained in an entire tree [22, 23], making it more useful for making comparisons between different sites. In the context of forest management, over time LVR could also reflect the effectiveness of forest restoration initiatives in northern hardwood forests.

To improve our understanding of the factors associated with the large-scale variation in wood quality in northern hardwoods, our aim in this study was to explore site-to-site variations in LVR for yellow birch and sugar maple in the forests of Quebec, Canada. These species were chosen both for their abundance in the North American temperate deciduous forests and for their economic importance. To pursue our objective, we first reassessed an existing lumber value model in order to make reliable estimates of LVR for trees contained in a large provincial forest inventory database. Next, using ‘ensemble’ methods, we developed a statistical model to link estimates of this indicator to site- and plot-level descriptors. Specifically, we used a combination of boosting and regression trees for data analysis and model prediction. These methods allowed us to include complex nonlinear interactions between predictors in the models. Finally, we used the predictions of LVR to produce ‘hotspot’ maps to visualize the variation in timber quality potential across the distribution range of sugar maple and yellow birch in the Province.

## Material and Methods

This study was conducted within the mixed and deciduous forests of Quebec, Canada. As no field work was involved in our study, no life form was put at risk during our work and no field permit was necessary. Temporary sample plot (TSP) data covering the period from 1991 to 2012 were provided by Quebec’s Ministry of Forests, Wildlife and Parks. To be included in the analysis, a plot needed to contain at least one yellow birch or one sugar maple tree with a diameter at breast height (DBH, 1.3 m above the ground) larger than 23 cm. This corresponds to the lowest merchantable diameter limit for sawlogs, in accordance with the hardwood tree grading system used in the province [24]. A total of 22579 400-m^2^ TSPs were selected, with the two species occurring concomitantly in 7331 of these. The assessment of LVR is organised in two main parts in the following sections. The first part illustrates how an existing method was reassessed and recalibrated to obtain lumber value estimates for individual trees in each TSP. The second part describes how stand and plot-level characteristics were used to predict LVR values at the landscape level.

### Reassessment of the lumber value estimation method

The lumber value of individual stems was estimated using a combination of predictive models and equations. As the first step of this process, the volume by log grade was estimated using the models developed by Fortin et al. [25] for yellow birch and sugar maple. These models can be applied to predict the occurrence and volume of different log grades in standing trees, as described by Petro and Calvert [26]. According to this classification, logs can be arranged into three categories, namely F1, F2 and F3, in descending order of quality [27]. A fourth grade (F4) was also included to account for the possibility of obtaining sawn wood from short logs [28, 29], i.e. those with a small-end diameter ranging from 16 to 20 cm and length from 1.2 to 2.4 m. The log grade and volume predictions are based on variables measured in Quebec’s forest inventory, namely tree species, DBH, and tree quality class. The latter is based on Monger’s [24] classification system, which is analogous to that developed by the US Forest Service for northern hardwoods [30]. Using a combination of stem DBH thresholds and the distribution and size of defects along the stem, trees are categorized into four classes, indicating their processing potential [7].

Once the volume by log grade was estimated for the TSP dataset, the second step consisted in using the method from Petro and Calvert [26] to estimate the lumber value contained in a log (LV) of a given quality class (Eq 1):

$$LV=QI\cdot P\cdot V_{sw}$$ (1)

where LV is lumber value ($US), *QI* is the quality index (see Eq 2), *P* is the market price for a class 1C (1 Common [21]) sawn board ($US m^-3^), and *V*_*sw*_ is the sawn wood yield of each log (m^3^), estimated from volume tables by the authors.

This method was created as a way to estimate the value of different log grades while accounting for fluctuations in current sawn wood prices. Petro and Calvert [26] chose the National Hardwood Lumber Association’s (NHLA) board class 1C as a market price reference, since it was the most important category for both supply and demand. Market prices for the class 1C sawn boards were presented from 1953 to 1972 in their study, when average prices for yellow birch varied from 65 to 83 $US m^-3^, and from 58 to 73 $US m^-3^ for sugar maple.

The *QI* indicates the relative value of a log in terms of yield of 25-mm-thick (1 inch) lumber pieces. It represents the sum of the sawn wood yield by board class multiplied by the relative price by board class (Eq 2):

$$QI=\sum \left(Y_{c}\cdot RP_{c}\right)$$ (2)

where *Y*_*c*_ is the sawn wood yield by board class (%) obtained by the authors in a sawmilling study, and *RP*_*c*_ is the ratio (dimensionless) between the market price for sawn wood of a given NHLA board class and the price for the reference grade (i.e. 1C).

Petro and Calvert [26] showed that the ratios between the market prices of each NHLA board grade were fairly constant through time. To assess the current applicability of the method, we used data from a sawing study conducted by Havreljuk et al. [7]. The database comprised 32 yellow birch and 64 sugar maple trees sampled from multiple stands in two regions of the province of Quebec. Trees were measured and categorized before being converted into boards. Independently of their dimensions, logs were sawn to maximize the production of high grade lumber (i.e. knot-free and sapwood) [31]. Boards were graded according to the NHLA standards [21] after being kiln dried. The board classes were later regrouped to match those described by Petro and Calvert [26], i.e. FAS, 1C, 2C and 3C. The board market prices for calculating *RP*_*c*_ were obtained from the Hardwood Market Report [32], averaged for a five-year period, from 2008 to 2012. Eq 1 was then used to calculate the LV.

**Fig 1:**
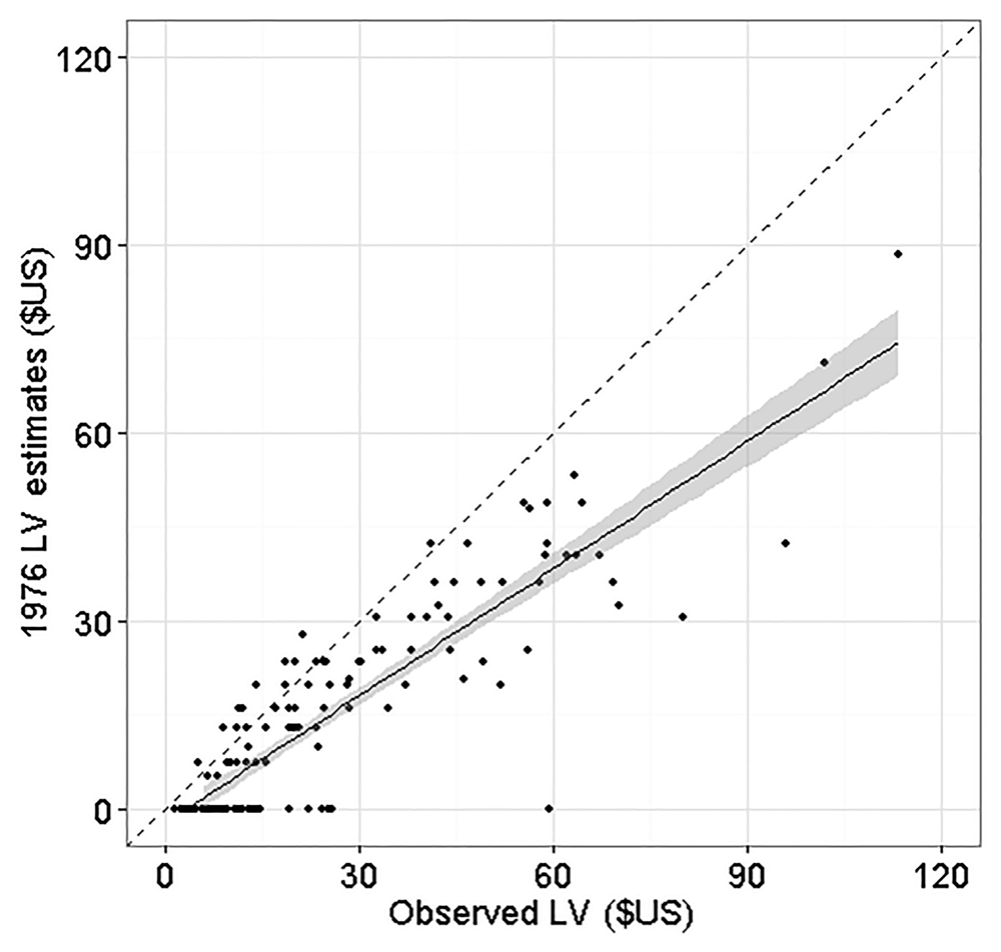
Comparison between LV predicted with parameters from 1976 and the observed LV. The black dashed line is the reference line, with slope of 1, and the shaded area represents the 95% confidence interval.

Even when the prices were updated to current values, the method proposed by Petro and Calvert [26] tended to underestimate the LV of individual trees (Fig 1). The main reason for this bias is the smaller variation in market price among the board quality classes observed by those authors compared to the current price variation (Table 1). The range of this variation directly influences the relative price (*RP*_*c*_), one of the key variables in the calculation of *QI*. Another factor that influenced the underestimation of LV estimates using Petro and Calvert [26]’s equations was the volume of sawn boards (*V*_*sw*_). The original method underestimated this volume for all log classes, possibly as a result of changes in sawing techniques that occurred over the past four decades. Furthermore, Petro and Calvert [26] did not consider the use of short logs for producing lumber. We hence considered that a reassessment accounting for current sawmilling practices and market conditions was necessary.

**Table 1:** Relative prices by NHLA [21] board class.

Board class	1976	2012^a^	
		Yellow birch	Sugar maple
FAS	1.44	1.81	1.65
1C	1.00	1.00	1.00
2C	0.71	0.70	0.69
3C	0.43	0.25	0.37

^a^ Averaged values from 2008 to 2012

After confirming that patterns of dispersion and variation in *QI* and *V*_*sw*_ were similar for both species, we then recalibrated the models for the two species combined. Because our intention was to propose a practical way of estimating LV, we tried to eliminate the use of proxy variables. Therefore, instead of simply reassessing *Yc* and updating *RPc* using current market values, we decided to simplify the whole concept by modeling *QI* as a function of gross log volume (*Vg*) and the volume for the various log classes (*V*_*F1*_, *V*_*F2*_, *V*_*F3*_ and *V*_*F4*_), variables that can be easily obtained (Eq 3):

$$QI=\left(\beta_{0}+\beta_{1}\cdot \frac{V_{F1}^{2}}{V_{g}}\right)+\left(\beta_{2}+\beta_{1}\cdot \frac{V_{F2}^{2}}{V_{g}}\right)+\left(\beta_{3}+\beta_{1}\cdot \frac{V_{F3}^{2}}{V_{g}}\right)+\left(\beta_{4}+\beta_{1}\cdot \frac{V_{F4}^{2}}{V_{g}}\right)$$ (3)

where V_F1_^2^, V_F2_^2^, V_F3_^2^, and V_F4_^2^ are the squared lumber volume in each log class and β_0_, β_1_, β_2_, β3, and β_4_ are the model parameters to be estimated.

Predictions of *V*_*sw*_ were a function of *V*_*g*_ (Eq 4).

$$V_{sw}=\gamma_{0}+\gamma_{1}\cdot V_{g}$$ (4)

where γ_0_ and γ_1_ are the model parameters to be estimated.

The adjusted *R*-squared values for the *QI* and *V*_*sw*_ predictive models were 0.50 and 0.97, respectively. Parameter estimates for both models are presented in Table 2.

**Table 2:** Parameter estimates and associated standard errors (SE) for *QI* and *V*_*sw*_ predictive models.

Model	Parameter	Estimates	SE
*QI* ^a^	β_0_	0.64	0.090
	β_1_	1.09	0.187
	β_2_	0.60	0.064
	β_3_	0.49	0.039
	β_4_	0.48	0.029
*V*_*sw*_ ^b^	γ_0_	-0.01	0.001
	γ_1_	0.58	0.007

^a^ *QI*, quality index;

^b^ *V*_*sw*_, sawn wood yield of a log.

**Fig 2:**
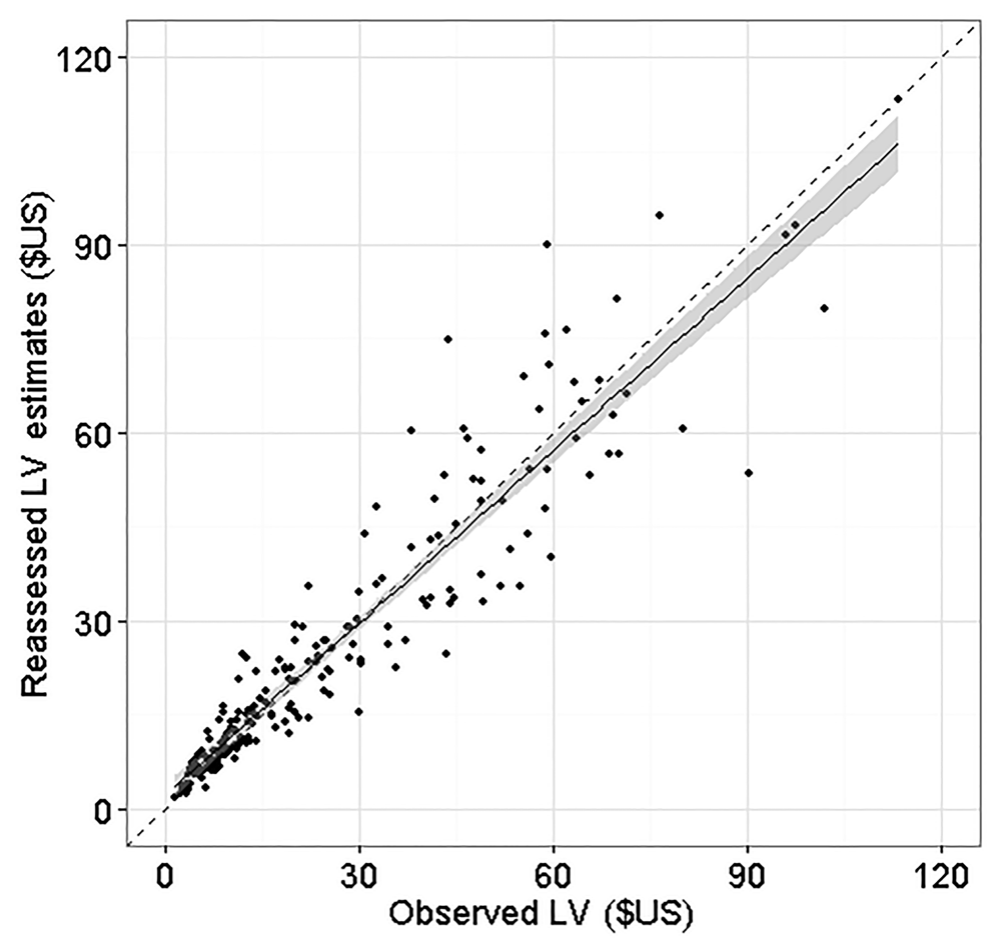
Comparison between LV predicted by the reassessed model and observed values. The black dashed line is the reference line, with slope of 1, and the shaded area represents the 95% confidence interval.

Once we had obtained predictions for *QI* and *V*_*sw*_, we then estimated LV using Eq 1. After the recalibration, the model provided unbiased estimates of LV (Fig 2).

### LVR predictions based on stand and site characteristics

The TSP database contained stand and site descriptors such as stand height class, age class, basal area, slope, altitude, stand density, ecological type, surficial deposit, and drainage class [33, 34]. All these variables were considered as potential predictors in a statistical model that aimed to predict LVR values. In all cases, the predictor variables excluded the characteristics of the subject trees, so that only the influence of the site and stand characteristics on LVR would be considered. The LVR for each tree was obtained by dividing the reassessed LV by the total roundwood volume of that tree.

The species and structural diversity indices were added to the database as potential predictor variables to describe the stand structure and composition. Both these indices were based on the exponential form of Shannon’s index (*H’*_*exp*_) [35], as follows:

$$H_{exp}^{\prime }=exp^{\left(-\sum_{i=1}^{s}p_{i}\cdot \text{ln}p_{i}\right)}$$ (5)

In the species diversity index, *p*_*i*_ was the proportion of occurrence of one species and *S* the total number of species. In the structural diversity index, *p*_*i*_ was the proportion of one DBH class and *S* the number of DBH classes. A value of zero for these indices indicated that there was no stand-level variability for the index in question.

To test if climatic conditions were associated with variations of LVR, data obtained using BioSIM [36], averaged for the period of 1981 to 2010, were tested along with the previously described variables. These included, for each plot location, the annual mean of daily minimum, mean and maximum temperatures (°C), annual total precipitation (mm/year), annual total snowfall (mm of water), annual mean of daily relative humidity (%), mean wind speed (km/h), growing season (days), annual potential evapotranspiration (mm), aridity (accumulation of monthly water deficit, mm), annual total radiation (MJ/m^2^), and number of days with precipitation.

The relationship between LVR and the limits of atmospheric acid deposition in the soil and its exceedance were also tested. The elements used to determine these limits are the maximum critical load of sulfur (*CL*_*max*_) deposition and its exceedance, both in molar equivalent for potential acidity (eq ha^-1^ yr^-1^). The maximum critical load was obtained based on the methodology described by Ouimet et al. [37] (Eq 6).

$$CL_{max}=BC_{dep}-Cl_{dep}+BC_{w}-B_{C_{u}}-Alk_{le\left(crit\right)}$$ (6)

where *BC*_*dep*_ is the sum of K, Ca, Mg and Na deposition rates, *Cl*_*dep*_ is the Cl deposition rate, *BC*_*w*_ is the soil weathering rate of K+Ca+Mg+Na, *B*_*cu*_ is the net K+Ca+Mg uptake rate, and *Alk*_*le(crit)*_ is the critical alkalinity leaching rate, all variables in eq ha^-1^ yr^-1^. The exceedance is the difference between the averages of total annual depositions of sulfur and the maximum critical load for years 1999–2002. The details on the *CL*_*max*_ and exceedance mapping for Quebec can be found in Ouimet and Duchesne [38].

#### Boosted Regression Trees

The statistical analyses were performed with the full set of stand, climatic and soil acidity variables as the predictors, and LVR as the response variable. This base LVR was obtained using the reassessed equations of Petro and Calvert [26]. Candidate models were developed and selected using Boosted Regression Trees (BRT), a machine-learning technique that produced a predicted LVR model based on an ensemble of decision trees. The BRT method combines two statistical techniques, namely boosting and regression trees. The latter is a technique that uses decision trees formed by binary splits to build a predictive model, taking into consideration the interactions between variables [39, 40]. Boosting uses a forward stage-wise procedure, where the regression trees are fitted iteratively to a subset of the training data [40]. These subsets are randomly selected without replacement, and the proportion of the training data (bag fraction) can be specified. This procedure, known as stochastic gradient boosting, introduces some randomness into the boosted model, improving accuracy and reducing overfitting [41]. Although BRT is normally used for predicting presence/absence data, the works of Moisen et al. [42], Carslaw and Taylor [43], and Carty [44], among others, have shown that it can be efficiently used for modelling continuous variables.

The analyses were made using the R statistical programming environment, version 3.1.2 [45], using the 'gbm' [46] and 'dismo' [47] packages. Separate models were developed for each species. Before fitting the models, the database for yellow birch and sugar maple was randomly split into training (70%) and validating datasets (30%). Several models were created to verify the combination of BRT parameters, namely the tree complexity (*tc*), learning rate (*lr*) and regression trees, that would result in the minimum predictive error. Those models were fitted to the training dataset and, once the best models were selected, the validating dataset was used for evaluating the model predictions.

The BRT method is capable of modelling complex variable interactions [43], by increasing the *tc*. This parameter was set either as 1 (indicating that no interactions among variables would be allowed), 2 (two-way interaction), 5, 7 or 10. The *lr*, which determines the contribution of each tree to the model, varied from 0.1 to 0.0001. Slower learning rates are normally preferred because they shrink the contribution of each decision tree, giving more reliable estimates of the response, but at the cost of increased computation time [48]. Faster learning rates may increase the predictive deviance too rapidly after reaching the minimum, indicating that the method is overfitting the final model. We chose a combination of parameters that would give us reliable predictions with the fastest computing time. For all models, we used a bag fraction of 0.5, meaning that, at each iteration, 50% of the data would be drawn at random, without replacement. The models were run and the results compared to determine the best combination of parameters. The predictive performance of the models was based on the proportion of the deviance explained (*D*-squared) [49], and on the root mean squared error (RMSE).

To evaluate the contribution of each term in reducing the overall model deviance and help eliminating non-influential variables [50], an index of relative importance was used. The relative importance of predictor variables was based on the number of times a variable was selected for splitting in the tree weighted by the squared improvement to the model as a result of each split [51]. The sum of the relative contributions was added to 100, and the contribution of each variable was scaled accordingly.

The nature of the relationship between the predictor variables and the response [52] was verified with the aid of partial dependence plots. Once the relevant predictors were selected, based on the change of predictive deviance, the model was then simplified to include only the variables that would contribute to the predictions.

#### Provincial-scale analysis of LVR estimates

The LVR estimates from the BRT method were plotted to maps and a hotspot analysis was performed using the Getis-Ord Gi* method [53] from ArcMap 10.1 [54]. This analysis was used to verify whether the predicted values were clustered, meaning that there would be areas with significantly higher and significantly lower LVR within the Province. The interaction between a value and its neighbours was set as a fixed distance band, where the scale of the analysis was constant across the study area [55]. The distance band for each species was determined by using the Spatial Autocorrelation (Global Moran’s I) method from ArcMap 10.1 [54], assuring a 99% likelihood of real clusters. For yellow birch, the distance was 40 km, and for sugar maple, 72 km. This procedure ensured that all features had at least one neighbour during the analysis and that all occurrences outside the fixed distance did not influence the TSP in question.

The clusters were originated by the combination of the z-score (standard deviation) and the p-value (probability of having a spatial pattern created by some random process). The resulting clusters were then categorized as either (i) very significantly higher/lower than mean (α = 0.01), (ii) significantly higher/lower than mean (α = 0.1), and (iii) mean.

## Results

### BRT model

A total of 35 base LVR predictive models per species were tested to verify the combination of parameters that would result in the minimum predictive error, with a stipulation that at least 1000 decision trees should be used for fitting the models. The learning rate and tree complexity values that gave the minimum predictive error for yellow birch were *lr* = 0.003 and *tc* = 10, while for sugar maple the values were 0.01 and 10, respectively. For yellow birch, the *D*-squared for the full model was 0.13 and the RMSE was 16.13, while for sugar maple the values were 0.17 and 17.79, respectively.

**Fig 3:**
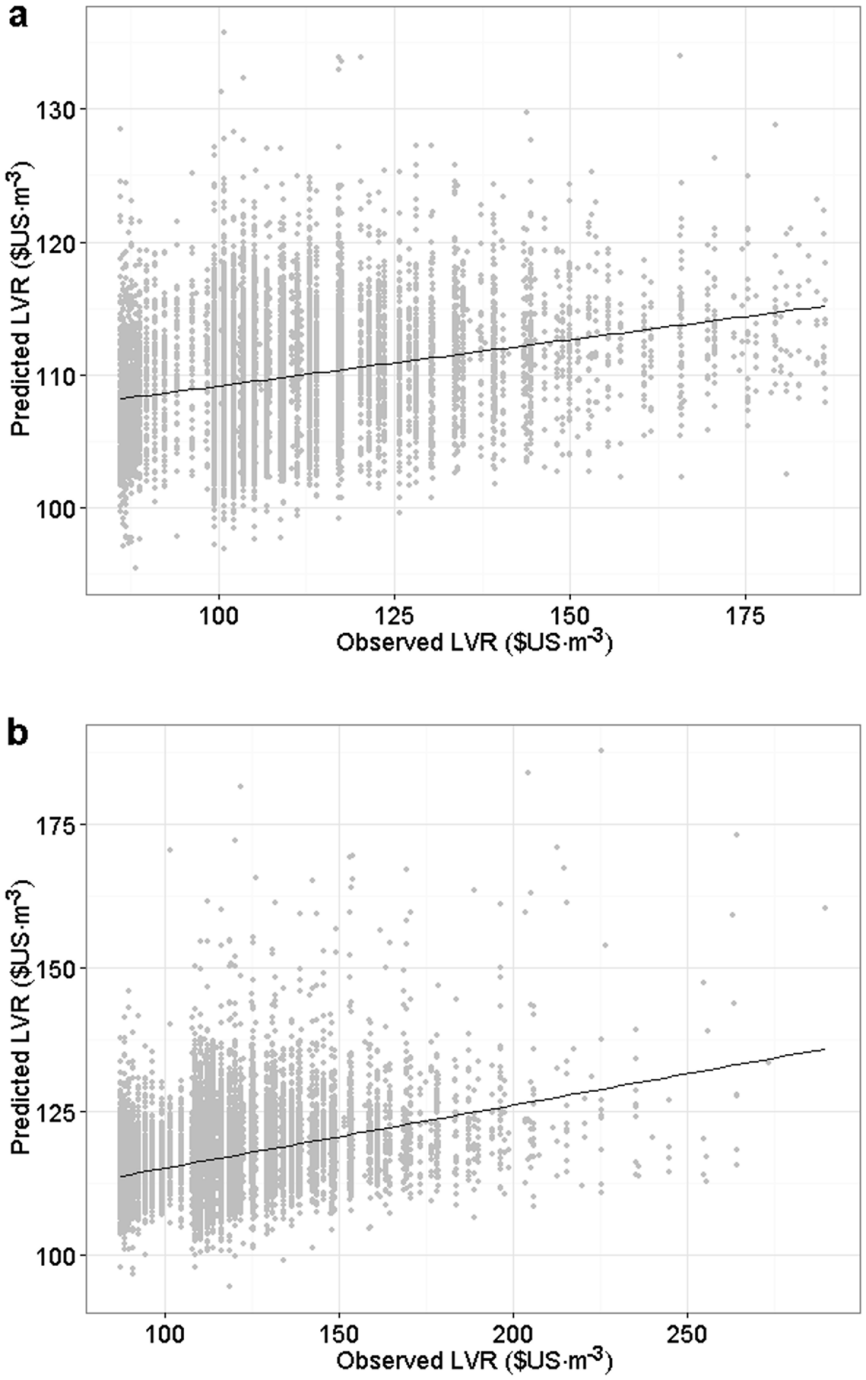
Comparison between predicted and observed LVR for (a) yellow birch and (b) sugar maple.

The relative importance of the predictors, along with the change of predictive deviance produced by BRT was used in the selection of the independent variables to simplify the final models. In these final models, *D*-squared for yellow birch remained 0.13 and the RMSE was 16.13. For sugar maple, the corresponding values were 0.18 and 17.16, almost the same as the values for the full model. A comparison between the predicted and observed LVR values performed to the validating dataset can be seen in Fig 3a and 3b.

#### Partial Dependence Plots

**Fig 4:**
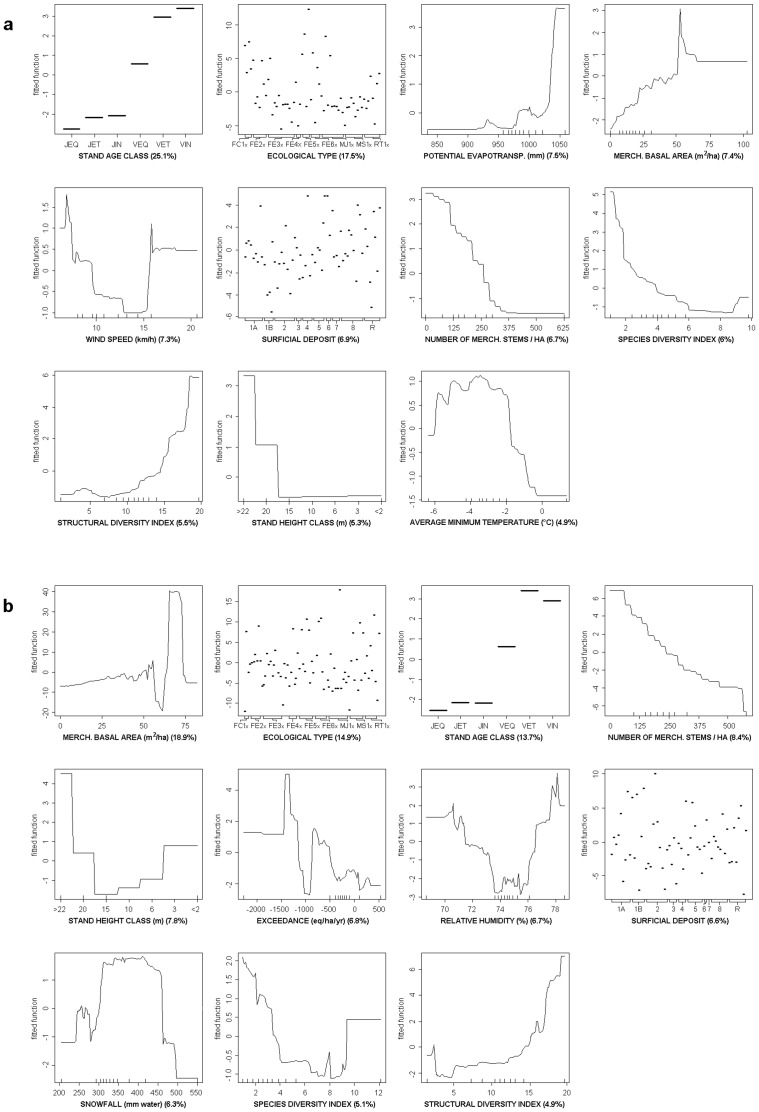
Partial dependence plots for each predictor retained in the final models for (a) yellow birch and (b) sugar maple. The respective relative importance for each independent variable is in parentheses. Note: Stand age class: JEQ—young even-aged stand, JET—young stratified stand, JIN—young uneven-aged stand, VEQ—old even-aged stand, VET—old stratified stand, VIN—old uneven-aged stand; Ecological types: FC1x —red oak, FE1x —sugar maple—bitternut hickory, FE2x —sugar maple—basswood, FE3x —sugar maple—yellow birch, FE4x —sugar maple—beech—yellow birch, FE5x —sugar maple—hop hornbeam, FE6x —sugar maple—red oak, MJ1x —yellow birch—sugar maple, MS1x —balsam fir—yellow birch, RT1x —hemlock; Surficial deposit: 1A —glacial deposits without specific morphology, 1B —glacial deposits characterized by their morphology, 2 —fluvioglacial deposits, 3 —fluvial deposits, 4 —lacustrine deposits, 5 —marine deposits, 6 —littoral marine deposits, 7 —organic deposits, 8 —slope and weathering deposits, R—rock substrate. A complete description of the ecological types and surficial deposit can be found at Berger and Leboeuf [34].

The partial dependence plots were obtained from term-wise plots of fitted functions versus observed values for the yellow birch and sugar maple final models (Fig 4a and 4b, respectively). Despite the partial dependence plots not being an exact representation of the relationship between the predictors and the explanatory variables, they can be useful for understanding the nature of these relationships.

For both species, LVR from fitting data was positively correlated with stand age class, stand height class, structural diversity index and merchantable basal area, but showed a negative correlation with the number of merchantable stems and the species diversity index. The ecological types associated with high LVR were FC10, FE13, FE50, FE51, FE55, FE61, MJ11P, MJ12P for yellow birch, and FE33P, FE40, FE43, FE45, FE53P, FE54, MJ18, MS12P, MS14, RT13, RT18 for sugar maple. Those higher LVR values for yellow birch occurred in areas with soil depths varying from medium to shallow with abundant outcrops. For sugar maple, sites with soil depth ranging from medium to deep deposits gave higher LVR values.

A positive correlation between potential evapotranspiration and LVR, and a negative correlation between the average minimum temperature and LVR were observed for yellow birch. There was a complex pattern of variability of LVR as a function of wind speed; LVR values tended to decrease before rising steeply for sites with mean wind speed ranging from 15 to 20 km h^-1^. From the entire range of snow water equivalent where sugar maple is found in the study region, only the middle of that range gave higher LVR values. A negative correlation between soil critical load exceedance of sulfur and LVR was observed only for sugar maple.

#### BRT model predictions and hotspot analysis

**Fig 5:**
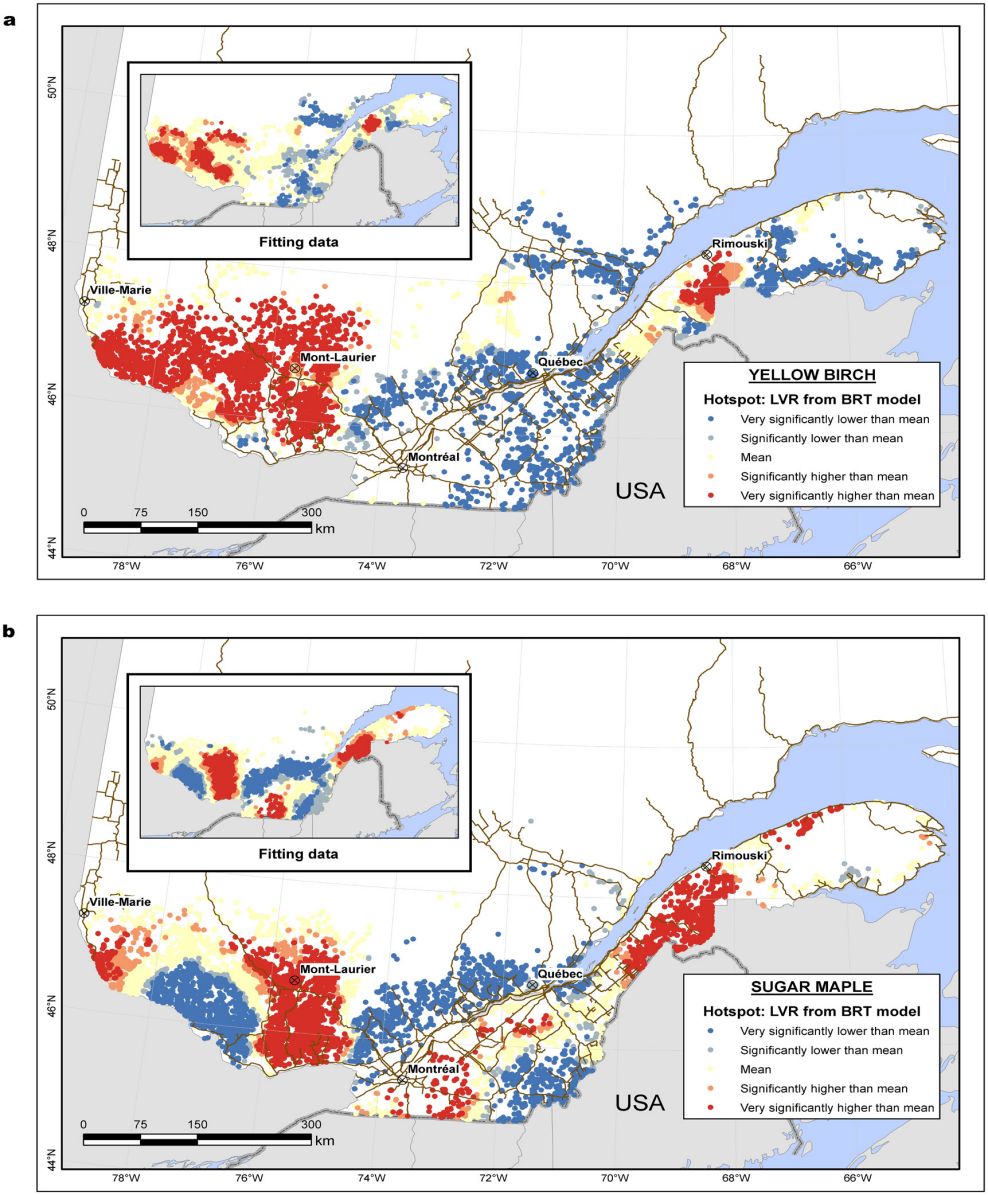
Distribution of LVR for (a) yellow birch and (b) sugar maple, across the province of Quebec. Comparison between LVR from fitting data (inset figure) and predicted LVR from BRT.

‘Hotspot' maps of the BRT model predictions on the validation dataset were plotted for yellow birch (Fig 5a) and sugar maple (Fig 5b), along with ‘hotspot’ maps performed to the fitting data for comparison. The presence of clusters on the maps indicates the existence of underlying spatial patterns. Even though the predicted values dispersion (μ = 109.9; σ = 4.5; range = 95.5–135.7 for yellow birch, and (μ = 116.9; σ = 6.3; range = 94.4–187.7 for sugar maple) differed from the fitting data dispersion (μ = 109.8; σ = 16.9; range = 86.1–281.7 for yellow birch, and μ = 117.0; σ = 18.5; range = 87.4–302.3 for sugar maple),the similarity between the pairs of plots suggests that most of the regional-scale variation in LVR was captured by the BRT model.

Clusters of statistically significant high values for yellow birch were observed in the west side of the province, as well as a small area south of Rimouski. Clusters significantly lower than the mean for the same species were found in the centre of the province, as well as east of Rimouski. For sugar maple, the high and low values occurred in several locations across the province; the regions surrounding Mont-Laurier, Montreal and south of Rimouski produced higher values, while east and far south of Quebec City, as well as southwest of Mont-Laurier presented lower values.

## Discussion

Due to the observed price gap between the most valuable and the least valuable board grades from 1976 and 2012, the original model from Petro and Calvert [26] was no longer providing a reliable, accurate estimate of LV. When *RP*_*c*_ was eliminated from the reassessed model and only the volume of each log class and the gross log volume were retained as predictors, the results were improved. In the original model, *V*_*sw*_ was obtained from volume tables. The use of a simple linear model with only one independent variable (*V*_*g*_) yielded more precise estimates for *V*_*sw*_, bringing the predictions closer to the observed values. The discrepancy in predicted *V*_*sw*_ between the models developed in this study and those of Petro and Calvert [26] can be largely explained by the inclusion of short logs, which implies that the recalibrated model predicts the maximum LV that could be extracted from a tree. The use of Fortin et al. [25]'s model to predict volume by log grade likely introduced additional correlated errors into the LV model. However, since these models were parameterized using different datasets, it was not possible to account for potential error propagation by simultaneously estimating the model parameters in a system of equations [56, 57]. In addition to being able to estimate LV using up-to-date parameters, the reassessed model could be a useful tool for future studies, in particular as an indicator of timber quality.

The two LVR predictive models produced by the BRT method presented low *D*-squared values. Despite the clear linear trend between the predicted and observed values, the relationship did not follow the 1:1 line because the range of predicted values was much smaller than that of observed values (Fig 3a and 3b). This was expected, considering that the LVR variation among trees of the same species is highly influenced by intrinsic tree characteristics [58]. Nevertheless, the final model results indicate that there is an underlying cause for LVR variation at a larger scale that was not due to individual tree characteristics, but more likely to its growing environment.

The variation in LVR attributed to stand and site characteristics was well captured by the BRT model. This can be visually confirmed by the hotspot analysis (Fig 5a and 5b), where the pattern of the fitting data and the BRT model predictions are very similar. Hence the use of BRT in conjunction with a hotspot analysis proved to be a useful tool for visualising large-scale variations in timber quality.

The observed relationship between LVR and stand and environmental characteristics suggests that sites providing the best growth potential for a species are also those where LVR values are highest [59–61]. Our results are in agreement with other studies where stand productivity has been positively associated with stand structural diversity [62, 63], and stand height [64]. However, these results could also reflect the fact that forests with more complex structures were probably not subjected to high-grading in the past, since more intensive cuts can result in simpler stand structures [65–67].

The inverse relationship between LVR and species diversity index indicates that trees were of a higher quality when stands were composed of fewer species. While a decline in stand productivity has been associated in some cases with higher species variability in mixed stands [68, 69], Paquette and Messier [70] observed the opposite trend, providing evidence for productivity gains with an increase in species diversity. In our study, the decrease in quality of the target species may be an indirect consequence of the improved growth of other species, such as American beech (*Fagus grandifolia* Ehrh.) [71, 72]. Alternatively, it may be that more diverse stands to the south of our study area, closer to densely populated areas, had been subjected to high-grading. In either case, further work is necessary in order to identify true causal links.

In our study, the pattern between ecological type and LVR was not clear. Some authors suggest a correlation between ecological types and tree growth [73, 74], but the tendency varies between species. In our study, most of the ecological types associated with high LVR corresponded to the hotspot areas in the provincial maps (Fig 5a and 5b). These ecological types did not necessarily have the highest mean DBH, which indicates that other factors influence LVR values.

Yellow birch can grow in areas with a variety of geology and soil types, being present on nutrient-poor, acidic, well-drained soils [75, 76]. Sugar maple grows well on moderately or well-drained, deep fertile soils, with glacial surficial material and moraines [75–78], especially if there is abundant organic matter [79]. Yellow birch appears to thrive in more typically boreal conditions than sugar maple, perhaps as a result of reduced competition from other species. Indeed, this species is known to grow better in cooler, more humid conditions [79], which were also found to be correlated with higher stem quality [12]. Equally, the negative correlation between LVR and average minimum temperature shows that this species also grows well in sub-boreal conditions. The negative correlation between wind speed and LVR may result from the fact that even slow wind speeds can be associated with a reduction in plant growth due to mechanical stimulation, increase of gaseous exchanges and water loss [80–84].

Even though the correlation between LVR and snowfall for sugar maple was not conclusive, it indicates that stands with the potential to produce higher quality timber coincide with the regions at the high and low ends of the snow accumulation range. Snowfall impedes the freezing of the root system and soil water, and contributes to the soil moisture during the following spring [85–87]. On the other hand, when the amount of snow is increased, the beginning of the growth season may be delayed. Lower or higher than average snowfall might then be linked with a decline in tree growth, which could result in lower LVR values.

The critical load exceedance may be responsible for the declining tree vigour and growth, and crown dieback of some hardwoods [88–90], especially for trees that need richer soils, such as sugar maple [79]. These depositions are known to change the chemical composition of soils through the depletion of plant nutrient cations, and increases in aluminium mobility and sulfur and nitrogen content [91]. The ‘cold’ spots for sugar maple on the provincial map coincide with areas with poor soils, with high exceedance of acidity, which is thought to be a cause of sugar maple decline and dieback [72]. In addition, the ‘cold’ spot in the west represents an area of poor growth for sugar maple where forests are often dominated by eastern white pine (*Pinus strobus* L.). On the other hand, the high LVR areas near Rimouski are located in less acidic soils having no exceedance of acidity [38]. Sugar maple trees from this region usually have light-coloured sapwood and fewer defects [11], characteristics that result in higher LVR values.

The hotspots for yellow birch in the West of the Province are located in areas where higher evapotranspiration is observed [92]. The ‘cold’ spot near Montreal and Quebec City coincides with a more densely populated area, with a high proportion of private forests. Although the history of management may have differed in forests under public and private ownership, the relative influence of land tenure type in comparison to environmental factors is uncertain. It could also be due to the fact that these areas have deeper, richer soils [92], which would benefit species other than yellow birch.

## Conclusions

In this study we used lumber value recovery (LVR) as an indicator of timber quality in two commercially important northern hardwood species. This allowed us to study the influence of stand and site characteristics on the current lumber quality throughout the province of Quebec. The interpretation of the results made it possible to identify potential links with either the intrinsic characteristics of the site or the effect of past management strategies. The interrelated nature of these factors, however, is likely to obscure the true causal relationships. For example, it is more likely that high-grading occurred in better sites, where higher profits could be made in a single entry [66].

Despite this uncertainty, our results strongly indicate that some sites have a higher potential for producing high quality timber than others. The factors associated with better timber quality were also the ones usually associated with higher productivity. Sugar maple seems to perform better in sites with deeper and richer soils, whereas for yellow birch, shallower soils and more sub-boreal climatic conditions appear to be more favourable. At the provincial level, there are some regions where lumber value is higher on average, so stands in these locations might be better suited to the implementation of restoration measures that favour the harvesting of low-vigour, defective trees. For sugar maple, soil acidity appears to be among the underlying factors that explain the regional variation. In such cases, restoration measures aimed at improving site conditions might be considered [72, 89]. Alternatively, placing more management emphasis on the production of high quality timber from other tree species, such as American beech, red oak (*Quercus rubra* L.) or eastern white pine, could be envisaged.
